# Antioxidant Activity of Deferasirox and Its Metal Complexes in Model Systems of Oxidative Damage: Comparison with Deferiprone

**DOI:** 10.3390/molecules26165064

**Published:** 2021-08-20

**Authors:** Viktor A. Timoshnikov, Lilia A. Kichigina, Olga Yu. Selyutina, Nikolay E. Polyakov, George J. Kontoghiorghes

**Affiliations:** 1 Institute of Chemical Kinetics & Combustion, 630090 Novosibirsk, Russia; timoshnikov@kinetics.nsc.ru (V.A.T.); lilkicha@gmail.com (L.A.K.); olga.gluschenko@gmail.com (O.Y.S.); polyakov@kinetics.nsc.ru (N.E.P.); 2 Postgraduate Research Institute of Science, Technology, Environment and Medicine, Limassol CY-3021, Cyprus

**Keywords:** deferasirox, deferiprone, antioxidant activity, iron chelation, copper chelation, Fenton reaction, lipid peroxidation, ascorbic acid, linoleic acid, dihydropyridine, NMR

## Abstract

Deferasirox is an orally active, lipophilic iron chelating drug used on thousands of patients worldwide for the treatment of transfusional iron overload. The essential transition metals iron and copper are the primary catalysts of reactive oxygen species and oxidative damage in biological systems. The redox effects of deferasirox and its metal complexes with iron, copper and other metals are of pharmacological, toxicological, biological and physiological importance. Several molecular model systems of oxidative damage caused by iron and copper catalysis including the oxidation of ascorbic acid, the peroxidation of linoleic acid micelles and the oxidation of dihydropyridine have been investigated in the presence of deferasirox using UV-visible and NMR spectroscopy. Deferasirox has shown antioxidant activity in all three model systems, causing substantial reduction in the rate of oxidation and oxidative damage. Deferasirox showed the greatest antioxidant activity in the oxidation of ascorbic acid with the participation of iron ions and reduced the reaction rate by about a 100 times. Overall, deferasirox appears to have lower affinity for copper in comparison to iron. Comparative studies of the antioxidant activity of deferasirox and the hydrophilic oral iron chelating drug deferiprone in the peroxidation of linoleic acid micelles showed lower efficiency of deferasirox in comparison to deferiprone.

## 1. Introduction

There are hundreds of thousands of iron overloaded patients belonging to different categories of inherited and other diseases, who receive regular red blood cell transfusions for the treatment of their refractory anemia [1,2,3]. Iron overload is considered as an independent adverse prognostic factor in all diseases. Excess iron from chronic transfusions is deposited in many organs causing serious damage and eventually death, unless effective chelation therapy is used for iron removal [4,5]. For example, most regularly transfused iron loaded thalassemia patients, not receiving chelation therapy, die before the age of 20 years because of congestive cardiac failure caused from excess, toxic iron deposition in the heart [1,4,5,6,7]. In addition, there are many other patients with normal iron stores such as Friedreich ataxia, Alzheimer’s and Parkinson’s disease patients, where localized “focal” iron deposits have been identified, by magnetic resonance imaging (MRI) techniques, as present in the brain, causing neurodegenerative damage [8,9,10,11]. The treatment of iron overload in regularly red-blood-cell-transfused patients and also patients with localized tissue “focal” iron deposits can only be achieved by the removal of toxic iron using chelation therapy [6,7,8,9,10,11].

There are currently three regulatory approved iron chelating drugs which are used daily for the treatment of transfusional iron overload, namely deferoxamine, deferiprone (L1) and deferasirox (DFRA) [4,6,7,8,12]. All three chelating drugs have different structures, physicochemical, pharmacological, toxicological and other properties [13,14]. Both L1 and DFRA are orally active, whereas deferoxamine is administered subcutaneously [4,6,7,8,12,13,14].

Investigations on the affinity and other interactions of the chelating drugs with essential and redox active metal ions are crucial for determining their therapeutic activity and toxicity potential [13,14,15]. Of particular interest are the interactions of chelating drugs with the essential transition and redox active metal ions iron and copper, which are present in all the cells and play vital role in physiological and biochemical pathways. Molecular studies using chelating drugs are very important because they can amongst other identify serious toxic effects including pro-oxidant effects and biomolecular damage, which under certain conditions can progress to subcellular, cellular and organ damage [16,17].

The molecular interactions with other metal ions in addition to iron and copper, such as zinc, and with nutrients such as ascorbic acid (Asc) and also many other natural or xenobiotic molecules, are also expected to interfere with the therapeutic and toxicity potential of iron chelating drugs as well as other drugs [16,17,18,19,20,21,22]. Furthermore, the treatment of many associated iron and copper metabolic imbalance conditions, as well as other diseases related to free radical pathology are also expected to be affected [23].

The study of the pro-oxidant/antioxidant effects of the iron chelating drugs and their iron and copper complexes is important for pharmacological/toxicological parameters affecting their mode of action and possibly their efficacy in vivo. However, the determination of the antioxidant potential of chelating drugs is also of major pharmacological interest because of possible therapeutic applications in many diseases associated with oxidative stress toxicity such as cancer, neurodegenerative, cardiac, liver, renal and other diseases [22,23,24,25,26,27,28]. Many studies have already been carried out investigating the antioxidant and other effects of L1 and deferoxamine in vitro, in vivo and in clinical models [29,30]. In contrast, the pro-oxidant/antioxidant effects of DFRA have not been yet been fully studied and characterized.

In this work the pro-oxidant/antioxidant effects of DFRA and its iron and copper complexes have been investigated using several molecular models of oxidative damage of linoleic acid (LA), Asc and dihydropyridine (DHP). In some of the studies L1 and Asc were used for comparison.

Linoleic acid peroxidation is widely studied in different model systems, including liposomes [31], but in most cases the rate of lipid peroxidation is estimated from the kinetics of formation of lipid peroxidation products [32,33,34,35]. Several other techniques have been previously used to estimate the initiation rate of lipid peroxidation, but in most cases these also relied on the detection of the products, for example, conjugated lipid hydroperoxides [36]. In the current investigation, we examined the influence of different ligands on the initial stage of lipid peroxidation, the abstraction of hydrogen atoms from bis-allylic position with formation of conjugated dienes and lipid radicals using ^1^H-NMR spectroscopy.

## 2. Results

**Figure 1:**
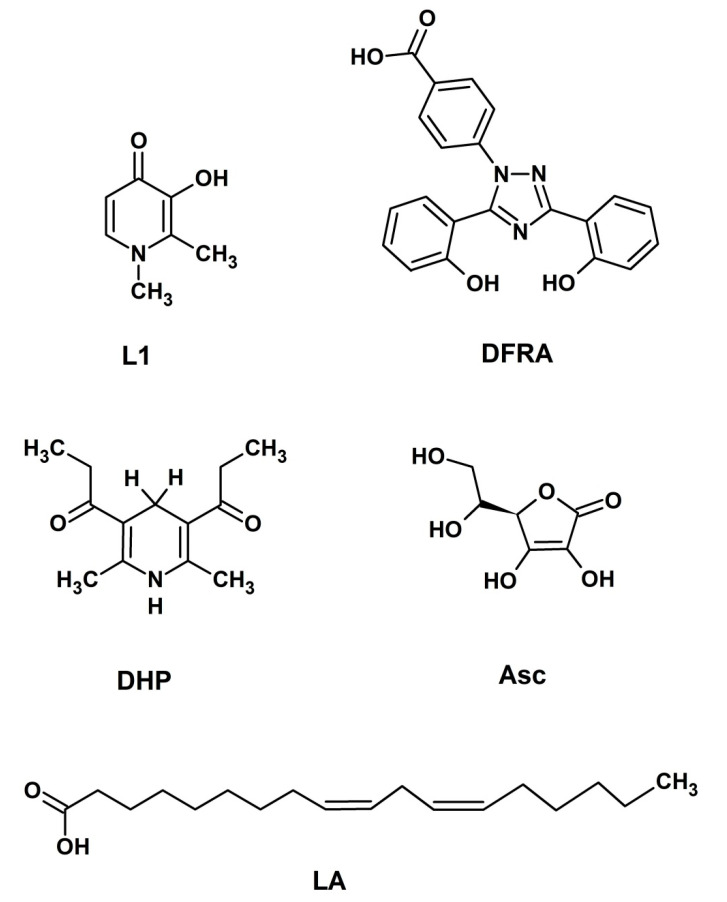
Chemical structures of 3-hydroxy-1,2-dimethylpyridin-4(1*H*)-one (deferiprone or L1), 4-[(3*Z*,5*E*)-3,5-bis(6-oxo-1-cyclohexa-2,-dienylidene)-1,2,4-triazolidin-1-yl]-benzoic acid (deferasirox or DFRA) (2R)-2-[(1S)-1,2-dihydroxyethyl]-3,4-dihydroxy-2H-furan5-one (ascorbic acid or Asc), (9*Z*,12*Z*)-octadeca-9,12-dienoic acid (linoleic acid or LA) and 1,1′-(2,6-dimethyl-1,4-dihydropyridine-3,5-diyl)-di(propan-1-one) (dihydropyridine or DHP).

Deferasirox is known to have high affinity for iron and copper binding. The stoichiometries of these complexes and stability constants have been measured in previous studies [37,38,39,40]. It was found that the complex of DFRA with Cu^2+^ ions has stoichiometry 1:1, and the stoichiometry of DFRA complex with Fe^3+^ ions 2:1. In the present studies we have tried to answer the question whether these complexes are redox active or not. For this purpose, the transition metal-induced oxidation of molecular model systems, namely LA, an unsaturated lipid, Asc and DHP as analogs of the nicotinamide adenine dinucleotide (NADH) molecule (Figure 1), have been studied by nuclear magnetic resonance (NMR) and ultraviolet-visible (UV-Vis) spectroscopy techniques.

### 2.1. UV-Vis Spectroscopic Studies of the Influence of Deferasirox on the Oxidation of Ascorbic Acid by Iron and Copper Ions

The first model for studying the antioxidant activity of DFRA and its chelate complexes with iron and copper ions was the oxidation of Asc. The biological functions of Asc include antioxidant, but also chelating activities [41,42,43]. In contrast to its antioxidant effects, Asc under certain conditions can also act as a pro-oxidant and a source of free radicals [41,42,44,45]. The mechanism of the pro-oxidant activity of Asc is related to its ability to reduce ferric iron via chelate complex formation, which results in the formation of ferrous iron and ascorbate radical. Taking into account that ferrous iron is involved in the process of reactive oxygen species (ROS) generation via the Fenton reaction, the reduction of ferric to ferrous iron will turn on the cyclic oxidation process. This is why the interaction of Asc with other chelators and their chelate complexes with Fe^3+^ and Cu^2+^ ions attracts great attention [46,47,48].

**Figure 2:**
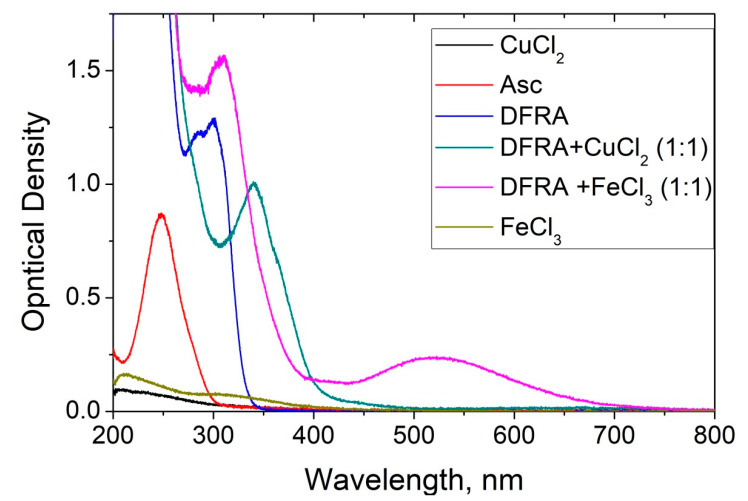
UV-Vis absorption spectra of deferasirox (DFRA, 0.1 mM), its chelate complexes with Fe^3+^ (0.1 mM) and Cu^2+^ (0.1 mM), FeCl_3_ (0.01 mM), CuCl_2_ (0.02 mM) and ascorbic acid (Asc, 0.1 mM) in ethanol solution.

A series of UV-Vis spectroscopic experiments were carried out to investigate the effect of DFRA on Asc oxidation in the presence of iron and copper ions at various ratios of the concentrations of chelator and metal ions in solution. The kinetics of changes in the optical density of the solutions were measured at 262 nm—a wavelength near the absorbance maximum in the absorption band of Asc (see Figure 2).

**Figure 3:**
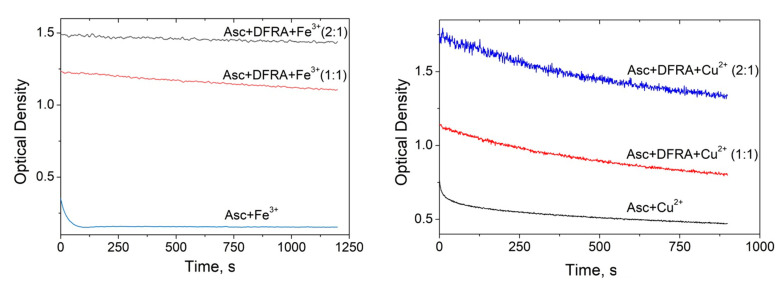
Changes in the rate of oxidation of ascorbic acid (Asc) in the presence of deferasirox (DFRA) Fe and Cu complexes. Time profile changes of the optical density of Asc at 262 nm (0.1 mM in EtOH) in the presence of FeCl_3_ (0.05 mM), and CuCl_2_ (0.02 mM) and various concentrations of DFRA (0, 0.05 and 0.1 mM for experiments with FeCl_3_, **left**), and DFRA (0, 0.02 and 0.04 mM for experiments with CuCl_2_, **right**).

The kinetics of oxidation of Asc in the presence and absence of DFRA iron and copper complexes were monitored in further UV-Vis spectroscopic studies. As it can be seen from Figure 3, the kinetics of the oxidation of Asc which correspond to the decrease in the optical density of the solutions are different in each case. In the case of mixtures containing iron ions, iron DFRA complexes and copper DFRA complexes, the kinetics follow a mono-exponential progression, reaching a plateau, while in the mixture containing copper ions, a rapid decrease first occurs, followed by a slow decay. It can be assumed that the primary process is due to Asc oxidation, while the secondary process is due to the decomposition of Asc oxidation products. Overall, it appears that the rates of these reactions are different and in each case depend on the type of transition metal involved.

When DFRA was added to the solution, the rate of Asc oxidation decreased significantly. The maximum effect of DFRA for Fe^3+^ and Cu^2+^ ions was achieved at a concentration ratio of DFRA:metal ion = 2:1. A further increase in the concentration of the chelator in the solution has practically no effect on the reaction rate. Based on these data, the rate constants of the reactions were calculated (Table 1). The rate constants of Asc oxidation have been determined using a mono-exponential rate model in the case of the iron ions, iron DFRA complexes and DFRA copper complexes. In the case of copper ions, the initial range of kinetics was selected, corresponding to the “fast” part, which is best described by the mono-exponential decay (about 75 s).

As it can be shown from the data described in Table 1, DFRA slows down the rate of Asc oxidation by more than 100 times in the case of Fe^3+^ ions and by about 30 times in the case of Cu^2+^.

### 2.2. NMR Studies of the Influence of Deferasirox and Deferiprone on Lipid Peroxidation of Linoleic Acid (LA) in the Presence of Iron and Copper Ions

The antioxidant effects of DFRA have been investigated in another molecular model of oxidative damage involving LA peroxidation in the presence of iron and copper ions. In these studies peroxidative changes in LA were monitored using an NMR method [49,50]. The reaction leading to LA peroxidation is widely used as a model of lipid oxidation [31,33,34,35]. This reaction consists of several stages. The “zero stage” is the generation of ROS via the Fenton reaction with the participation of iron and copper ions (1) and (2) as follows:

Fe^2+^ + H_2_O_2_ → Fe^3+^ + OH**·** + OH^−^ (1)

Cu^+^ + H_2_O_2_ → Cu^2+^ + OH**·** + OH^−^, (2)

In these reaction processes, OH radicals (OH**·**) are formed, which, in turn, are capable of conversion to other types of ROS, including the peroxyl radical and superoxide anion. The next stage of these reaction processes is “initiation”, in which the LA radical and the LA peroxyl radical are formed (3) and (4) as follows:

LH + OH**·** → L**·** + H_2_O (3)

L**·** + O_2_ → LOO**·** (4)

Later, at the stage of “prolongation”, LA radicals are also formed, as well as other reaction products, including aldehydes, hydroperoxides, and epoxides (5) and (6) as follows:

LOO**·** + LH → LOOH + L**·** (5)

LOOH → LO**·** → epoxides, hydroperoxides, aldehydes (6)

At the “termination stage”, various LA dimers and polymers are formed (7)–(9) as follows:

L**·** + L**·** → L-L (7)

LOO**·** + L**·** → LOOL (8)

LOO**·** + LOO**·** → LOOL + O_2_ (9)

**Figure 4:**
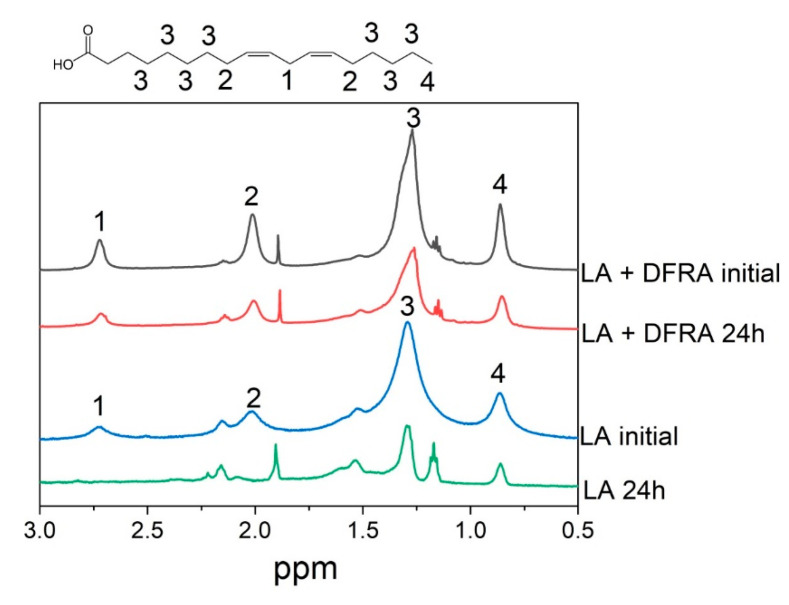
Chemical structure of linoleic acid (LA) and fragments of ^1^H-NMR spectra of the initial LA (3.5 mM) and the reaction mixture with H_2_O_2_ (0.5 M) and FeSO_4_ (0.1 mM) after 24 h in the absence and in the presence of deferasirox (DFRA, 2 mM). The studies were carried out at room temperature, in buffer solution (0.1 M) of pH 7.4. LA protons and their corresponding signals in the fragments of ^1^H-NMR spectra are designated by numbers 1–4.

It is also widely known that LA forms micellar solutions in an aqueous environment [51]. In this context and in order to study the kinetics of LA oxidation, a series of NMR experiments were carried out with the participation of LA micelles and iron and copper salts in the presence and absence of DFRA. Figure 4 for example, shows fragments of ^1^H-NMR spectra of the initial LA in the presence of ferrous sulfate (FeSO_4_) and after 24 h in the absence and in the presence of DFRA. It can be seen that after 24 h, the total signal intensity of LA protons was reduced significantly. In addition, the lines at 2.7 and 2.09 ppm, corresponding to protons near the double bonds (1 and 2), disappeared. All of the above, together with the fact that an insoluble precipitate was formed during the reaction, indicates the complete consumption of LA in the solution and the formation of polymers. In the presence of DFRA, 24 h after mixing, the overall signal level was also reduced, indicating the formation of polymers, but the signals corresponding to protons near double bonds did not completely disappear. This observation suggests that there is a decrease of the initiation rate of LA oxidation in the presence of DFRA.

It is known that the reaction of LA with OH radical starts with hydrogen atom abstraction from position 1 (see Figure 4) [31], and this observation was used in the present study for direct measurements of the rate constant of the “initiation” stage of the reaction. On the other hand, the decrease of total integral intensity on NMR signal due to polymers formation can be used to calculate the rate constants of the “termination” stage.

**Figure 5:**
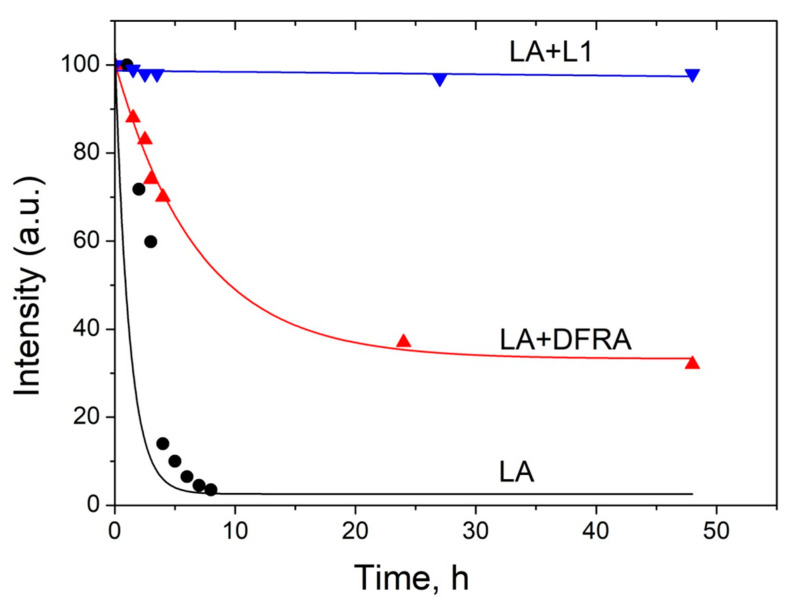
Kinetics of the initiation reaction phase of the linoleic acid (LA, 3.5 mM) peroxidation induced by FeSO_4_ (0.1 mM) and H_2_O_2_ (0.5 M) in the presence and absence of deferasirox (DFRA, 2 mM) and deferiprone (L1, 2 mM). The studies were carried out at room temperature, in buffer solution (0.1 M) of pH 7.4. The graphs were plotted using the decay of the integral intensity signal of the LA protons at 2.7 ppm (a.u., arbitrary units).

This molecular model of oxidative damage monitoring using LA peroxidation has been previously used for studying the antioxidant activity of the iron chelating drug L1 in the presence of copper ions [50]. Similar conditions have been used in a number of experiments for studying the time dependences of the integral intensity of the LA protons 1 and 4 (Figure 4), which were measured in the absence and presence of chelators. A typical example of a signal intensity change at 2.7 ppm is shown in Figure 5. The integral intensity of the initial signal at 2.7 ppm was normalized to 100. Intensity is given in arbitrary units (a.u.).

As can be shown in Figure 5, the rate of LA peroxidation caused by ferrous ions is significantly reduced in the presence of the chelating drugs L1 and DFRA. Furthermore, it can also be shown in this molecular model that the antioxidant activity of L1 in this LA peroxidation reaction is higher than that of DFRA.

**Figure 6:**
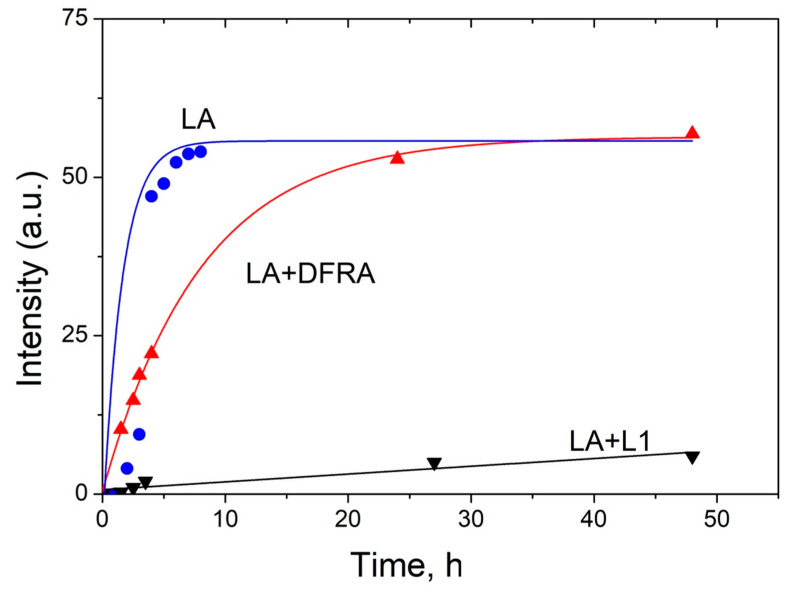
Kinetics of the termination reaction step of the linoleic acid (LA, 3.5 mM) peroxidation leading to polymeric products formation, which are induced by FeSO_4_ (0.1 mM) and H_2_O_2_ (0.5 M) in the absence and in the presence of deferasirox (DFRA, 2 mM) and deferiprone (L1, 2 mM). The studies were carried out at room temperature, in buffer solution (0.1 M) of pH 7.4. The graphs have been plotted using the decay of the integral intensity signal of the LA protons at 0.9 ppm (a.u., arbitrary units).

The kinetics of the formation of polymeric products of LA during the oxidation reaction with ferrous ions, which are calculated from the changes in the signal intensity of CH_3_ protons in LA, are shown in Figure 6. The procedure for data gathering and evaluation were based on previous observations, which suggested that a decrease in the integral intensity of the signal from the methyl group corresponds to an increase in the amount of various polymers formed in the oxidation reactions (7)–(9). The intensity of polymer products was calculated as follows (10):

I(t) = 100 − I_CH3_(t) (10)

where I(t) is the intensity of polymer products, I_CH3_(t) is the integral intensity of the signal from CH_3_-groups of LA, which corresponds to the concentration of LA. The integral intensity of the initial signal of the signal from CH_3_-groups at 0.9 ppm (I_CH3_(0)) was normalized to 100. Intensity is given in arbitrary units (a.u.).

As can be shown in Figure 6, the rate of formation of polymer products decreases significantly in the presence of L1 and DFRA chelators. In particular, in the presence of DFRA, the chain termination polymerization step is limited by the initiation step. In contrast, in the absence of a chelator, a faster reduction in the number of double bonds occurs with the formation of oxidized products that are already involved in the formation of polymers.

The experimental points in Figure 6 were approximated to fit an exponential decay, and subsequently the observed reaction rate constants were calculated from the fitting parameters. The calculated reaction rate constants are shown in Table 2.

The results presented in Table 2 suggest that DFRA inhibits LA peroxidation in the presence of iron and copper ions by 6 and 3 times, respectively, while the inhibitory effect of LA peroxidation by L1 under similar conditions is about 500 times. It is known that the redox potentials of DFRA and L1 chelate complexes with iron are very close [52], this is why it can be assumed that the reason of the observed antioxidant activity differences in this reaction might be due to differences in the lipophilicity of these two chelators. It is also known that L1 and its iron chelate complexes are hydrophilic and can be located outside the LA micelles. On the other hand, DFRA and its iron chelate complexes are lipophilic and can penetrate into lipid particles directly to the main reaction center (hydrophobic tail) [53].

### 2.3. NMR Studies of the Interaction of Deferasirox with Dihydropyridine

An additional molecular model system for studying the antioxidant activity of DFRA was the oxidation of DHP. The reactions involving DHP are often used as models of radical processes of electron and proton transfer in living systems with the participation of NADH [54,55]. Dihydropyridine was chosen for the studies because is capable of reacting with various radicals, including the OH radical.

**Figure 7:**
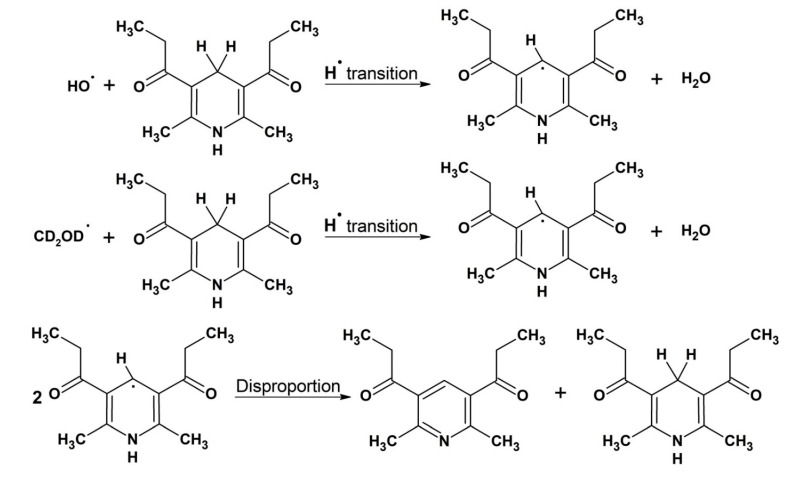
Scheme of the oxidation of 1,1′-(2,6-dimethyl-1,4-dihydropyridine-3,5-diyl)di(propan-1-one) (DHP). DHP reacts with a hydroxyl radical or ^●^CD_2_OD C-centered radical to form a DHP neutral radical. Subsequently two DHP radicals may be involved in the disproportionation reaction to form the initial DHP and 1,1′-(2,6-dimethylpyridine-3,5-diyl)-di(propan-1-one).

The formation of radicals for the NMR studies was initiated by the Fenton reaction using both iron and copper as catalysts and under similar conditions reported in previous studies [50,56]. Since the DHP is not soluble in water, the reaction was carried out in methanolic solution. In this case, the OH radical first reacts with the solvent CD_3_OD producing ^●^CD_2_OD C-centered radical [57]. As a result of hydrogen abstraction reaction by this radical, a neutral C-centered DHP radical is formed [54]. The main reaction product 1,1′-(2,6-dimethylpyridine-3,5-diyl)-di(propan-1-one) is formed via disproportionation reaction of two DHP radicals (Figure 7).

**Figure 8:**
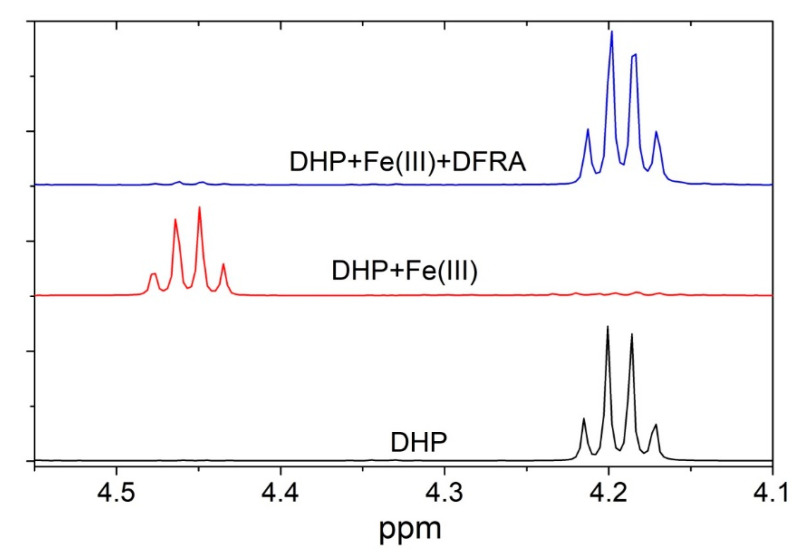
Fragments of ^1^H-NMR spectra of the initial DHP (5 mM) and of the reaction mixture with FeCl_3_ (0.2 mM) and H_2_O_2_ (0.2 mM) after 15 h in the absence and in the presence of DFRA (2 mM) in CD_3_OD solution at 278 K.

The antioxidant activity of DFRA in the molecular model of the oxidation reaction of DHP was studied in a series of ^1^H-NMR experiments, which were performed in the presence of iron and copper ions. Differences were observed in the ^1^H-NMR spectra of different reaction mixtures. Figure 8 for example shows fragments of the ^1^H-NMR spectra of pure DHP, as well as reaction mixture of DHP with Fe^3+^ ions in the presence and absence of DFRA, 15 h after the initiation of the reaction.

It appears from Figure 8 that DFRA almost completely inhibits the oxidation reaction of DHP with free radicals, which are caused by iron ions and hydrogen peroxide. Similar experiments were carried out with the participation of copper ions and hydrogen peroxide. The samples were kept at a temperature of 278 K in the absence of light. The fractions of degraded DHP were measured 15 h and also 2.5 days following mixing and the estimated amount of oxidation was based on the changes in the integral signal intensity of DHP protons. The fraction of degraded DHP in the absence of ferric ions was also calculated for comparison. The results are shown in Table 3.

The results in Table 3 show that in the presence of DFRA, the rate of DHP decomposition induced by iron and copper ions significantly decreases. These findings suggest that in non-aqueous media there is redox inactivity of chelate complexes of DFRA with iron and copper ions in the Fenton reaction. It is also worth noting that the yield of the reaction products in the case of the DFRA-Fe complex is lower than that of DFRA-Cu complex, which may suggest that there is a higher affinity of the chelator for iron under these experimental conditions.

## 3. Discussion

The experimental findings of this study have several important pharmacological, biological and physiological implications, especially for understanding the influence of DFRA on various redox systems. Deferasirox manifests its antioxidant activity in all studied model systems, including oxidation of Asc, peroxidation of LA micelles, and oxidation of DHP, with reduction in the rate of oxidation by up to several tens of times. Comparative analysis of the antioxidant activity of DFRA and L1 in lipid peroxidation showed a lower efficiency of DFRA in comparison to L1. The significance of these processes is underlined below from the different experiments carried out within this study.

Taking into account that in living systems copper and iron ions are mainly in bound forms, the study of the interaction of chelating drugs with natural complexants is an important task for elucidating the mechanism of drug action. One of the main natural antioxidant with chelating properties is Asc [42,43,45,58]. At the moment, there are many studies, both in vivo and in vitro, devoted to the interaction of chelating drugs with Asc [48,56]. However, this issue has not been studied for DFRA. It is known that Asc is capable of forming short-lived chelate complexes with transition metals, such as Fe^3+^ and Cu^2+^, which decompose to form Fe^2+^ or Cu^+^, and the ascorbate radical (11) and (12) [58,59,60]:

Fe^3+^ + AscH^−^ → Fe^2+^ + AscH· (11)

Cu^2+^ + AscH^−^ → Cu^+^ + AscH· (12)

Subsequently, ascorbate radicals are also capable of oxidizing, forming dehydroascorbate, which, in turn, undergoes the stages of hydration and decarboxylation, forming secondary reaction products [61,62]. In addition, the reduced forms of iron and copper ions can react with dissolved oxygen or another oxidizing agent, thereby forming additional channels for the oxidation of ascorbic acid (13) and (14) [52,63]:

Fe^2+^ + O_2_ → Fe^3+^ + O_2_**^·1^** (13)

Cu^+^ + O_2_ → Cu^2+^ + O_2_**^·1^** (14)

The study of Asc oxidation was carried out in ethanol since DFRA and its complexes with iron and copper ions have much lower solubility in water. Based on Figure 3 and Table 1, we can conclude that DFRA exhibits antioxidant activity in the reaction of Asc oxidation, with the rate constants of oxidation decreasing tens of times. In addition, it should be noted that in the case of iron ions, iron complexes, and complexes with copper, the limiting stage of the reaction is the stage of oxidation of Asc to the ascorbate radical.

Another, but no less important, effect of the antioxidant activity of chelators is the ability to inhibit lipid peroxidation. The study of this reaction is part of the solution to a wider problem, namely, the study of ferroptosis-programmed cell death. This mechanism is based on the oxidation of the cell membrane by ROS, formed in redox reactions with the participation of iron ions [64,65]. At this moment, the process of ferroptosis is quite well studied; however, the problems associated with radical reactions occurring in the lipid bilayer and the influence of various antioxidants, including chelators, on the reaction mechanism remain unsolved [66]. The effect of the chelator L1 on the ferroptosis process is best studied. Deferasirox and L1 are similar chelators. At the moment, these drugs are among the most widely used in clinical practice for the treatment of iron overload diseases [53]. Within this context, it was interesting to compare their effect on the system of lipid peroxidation with the participation of redox active transition metal ions.

At the moment, there are many different methods for studying lipid peroxidation, the vast majority of which are based on the detection of oxidation by-products of trichloroacetic and 2-thiobarbituric acids [33,34,67,68]. The NMR stands out positively in these methods, which makes it possible to follow the functional groups and determine the change in the concentrations of both the initial substances and the reaction products. It has been shown that DFRA inhibits the process of ferroptosis, as well as the reaction of lipid peroxidation in cell cultures, however, the antioxidant mechanism of action at the molecular level has not yet been described [64,69,70].

In the present study it was shown that DFRA inhibits LA peroxidation in micelles; however, the antioxidant effect of this chelator is significantly lower than that of L1. This finding can be explained by two possible mechanisms. One mechanism is that L1 oxidizes faster than DFRA, ferrous to ferric iron and forms ferric iron complex, which is not facilitating LA peroxidation. The other mechanism is related to the much higher lipophilicity of DFRA [53], which allows DFRA, as well as its chelate complexes to penetrate into micelles directly to the reaction center. Due to the reversibility of the complexation reaction, chelate complexes can decompose with the release of a free metal ion. As a result, a free metal ion is able to react with hydrogen peroxide to form a short-lived hydroxyl radical (1) and (2). In addition, it can be noted that in the case of DFRA, the rate of peroxidation is limited by the initiation stage (Figure 6).

The last part of our study was to investigate the effect of DFRA on redox active transition metal induced DHP oxidation. Dihydropyridines are widely used as analogues of NADH, which is a natural substrate in metabolic reactions of electron transfer in living cells [71,72]. Uncontrolled oxidation of NADH can negatively affect cell homeostasis and lead to cell death. As a result of this study, it was shown that DFRA inhibits the oxidation of DHP in a reaction involving Fe^3+^ and Cu^2+^ ions. It can be seen that the efficiency of the antioxidant activity of DFRA is higher in the case of Fe^3+^ than with Cu^2+^ which is explained by the higher affinity of the chelator for iron ions than for copper ions [37,38].

The antioxidant activity of DFRA in all three molecular model systems, confirms similar antioxidant activity observed in these and other models of oxidative damage with the other two chelating drugs L1 and deferoxamine [50,56,73,74,75].

## 4. Materials and Methods

### 4.1. Materials

Ascorbic acid (Asc, 99%), ferric chloride (FeCl_3,_ 97%), ferrous sulphate (FeSO_4_, 99%), copper chloride (CuCl_2_, 99%) and H_2_O_2_ (35.5%) were obtained from Sigma-Aldrich, Moscow, Russia. Deferiprone (L1; 99%) was received from LIPOMED Inc., Arlesheim, Switzerland. Deferasirox (DFRA; 99%) was obtained from Shanghai Daeyeon Chemicals Co., Ltd., Shanghai, China, DHP 1,1′-(2,6-dimethyl-1,4-dihydropyridine-3,5-diyl)-di(propan-1-one) (99%) was synthesized according to the classic Hantzsch scheme and obtained from prof. G. Duburs, Institute of Organic Synthesis, Riga [54]. All compounds were used as received. All experiments were carried out at room temperature.

### 4.2. Methods

#### 4.2.1. UV-Vis Optical Density Investigation of Ascorbic Acid Oxidation

UV-Vis optical density spectra and kinetics were measured in ethanol solution in 1 cm quartz cuvette using a SF-2000 spectrophotometer (Saint-Petersburg, Russia). The influence of DFRA on the oxidation of Asc was studied by addition of various concentrations of chelator into the mixture of iron or copper salts (CuCl_2_ and FeCl_3_) with Asc (0.1 mM) at room temperature and by monitoring changes at the absorption spectra. The wavelength of 262 nm, which corresponds to the absorption band of Asc, was selected based on the UV-Vis spectrometry data and the analysis of the spectra. This wavelength was selected because it also corresponds to a local minimum in this region of the absorption spectra of DFRA chelate complexes with iron and copper ions. The concentrations of the reagents used in the study were selected in the way that the optical density of the solution did not exceed the detection limit (optical density = 2) of the UV-Vis spectrometer.

#### 4.2.2. The ^1^H-NMR Study of Lipid Peroxidation

The study of lipid peroxidation was carried out using a reaction mixture consisted of LA micelles (3.5 mM), H_2_O_2_ (0.5 M), DFRA or L1 (1 mM), and FeSO_4_ or CuCl_2_ (0.1 mM) in deuterated phosphate-saline buffer (0.1 M) (pH 7.4). The reaction was initiated by addition of hydrogen peroxide. In the case of DFRA, LA was mixed with DFRA in chloroform, then the solvent was evaporated leaving behind a film, which was dissolved in deuterated phosphate-saline buffer (pH 7.4). Then FeSO_4_ or CuCl_2_ was added and the sample was incubated for 30 min to establish equilibrium. In the case of L1, LA was dissolved in chloroform, then the solvent was evaporated and the remaining film was dissolved in deuterated phosphate-saline buffer (pH 7.4). Then L1 and FeSO_4_ or CuCl_2_ was added and the sample was incubated for 30 min. The reaction was initiated by addition of hydrogen peroxide. The ^1^H-NMR spectra were recorded using a Bruker AVHD-500 spectrometer (500 MHz) (Rheinstetten, Germany).

#### 4.2.3. The Study of Dihydropyridine Oxidation

The investigations of dihydropyridine were carried out using a reaction mixture which consisted of 1,1′-(2,6-dimethyl-1,4-dihydropyridine-3,5-diyl)di(propan-1-one) (DHP) (5 mM), H_2_O_2_ (0.2 M), DFRA (2 mM), and FeCl_3_ or CuCl_2_ (0.2 mM) in deuterated methanol. The reaction was initiated by the addition of the metal salt into the sample. Dihydropyridines are quite sensitive to external influences, such as light [76], therefore samples were incubated at a temperature of 278 K and in the absence of light in order to minimize the effect of DHP autoxidation. The ^1^H-NMR spectra were also recorded using a Bruker AVHD-500 spectrometer (500 MHz).

## 5. Conclusions

Molecular studies including the redox effects of DFRA and its metal complexes with iron and copper are of major importance for determining the general pharmacological and toxicological properties of DFRA, as well as all other drugs. Furthermore, the findings from such studies may have therapeutic implications for patients receiving DFRA and other drugs.

In this study several molecular model systems of oxidative damage on Asc, LA micelles and DHP caused by iron and copper catalysis have been used for examining the effects of DFRA. The antioxidant activity of DFRA in the three molecular model systems of oxidative damage in this study, confirms previous findings in similar models and also the general antioxidant potential in biological systems of chelating drugs including DFRA, L1 and deferoxamine. However, differences remain between the chelating drugs on redox properties, affinity for metal ions including iron and copper, pharmacological and toxicological properties and also therapeutic effects.

Further studies are needed to investigate the antioxidant effects of DFRA and the other iron chelating drugs in different in vitro, in vivo and clinical models of oxidative damage. It is hoped that the antioxidant properties of DFRA and other chelating drugs will increase the prospects for the development of antioxidant drugs for the treatment of many different categories of free radical pathology patients, including patients with neurodegenerative diseases, cancer and cardiac, renal, liver and other organ damage.

## Figures and Tables

**Table 1:** The rate constants of the reactions of ascorbic acid (Asc) with Fe^3^^+^ and Cu^2+^ ions and their deferasirox (DFRA) complexes calculated from data from Figure 3. The concentration of Asc in both cases was 0.1 mM.

Metal Ions	Concentration of Metal Ions, ×10^−5^ M	Concentration of DFRA, ×10^−5^ M	k, M^−1^ s^−1^
Fe^3+^	5	0	832 ± 1
	5	5	7.6 ± 0.9
	5	10	7.0 ± 0.6
Cu^2+^	2	0	2808 ± 144
	2	2	86 ± 1.0
	2	4	82 ± 2.25

**Table 2:** Initiation and termination rate constants of the reactions of linoleic acid (LA) peroxidation induced by iron and copper ions in the absence and in the presence of deferasirox (DFRA) and deferiprone (L1).

Effect of Iron Ions	Initiation Rate Constant, ×10^−5^ s^−1^	Termination Rate Constant, ×10^−5^ s^−1^
LA	24.0 ± 1	17.0 ± 2
LA + DFRA	3.8 ± 0.5	3.5 ± 0.2
LA + L1	0.05 ± 0.01	0.07 ± 0.03
**Effect of Copper Ions**		
LA	10.0 ± 2 *	4.2 ± 0.8 *
LA + DFRA	3.0 ± 1	13.0 ± 5
LA + L1	0.02 ± 0.01 *	0.03 ± 0.01

* Data obtained from [50].

**Table 3:** The amount of oxidation products of DHP after the reaction with metal ions in the presence of hydrogen peroxide.

	After 15 h	After 2.5 Days
DHP	<1%	4%
DHP + Cu^2+^	57%	84%
DHP + Cu^2+^ + DFRA	9%	14%
DHP + Fe^3+^	96%	>99%
DHP + Fe^3+^ + DFRA	5%	7%

## Data Availability

Data is contained within the article.

## Sample Availability

Samples of the compounds are not available from the authors.

## Abbreviations

Asc	ascorbic acid
a.u.	arbitrary units
DFRA	deferasirox
DHP	dihydropyridine
H_2_O_2_	hydrogen peroxide
LA	linoleic acid
L1	deferiprone
MRI	magnetic resonance imaging
NADH	nicotinamide adenine dinucleotide
NMR	nuclear magnetic resonance
UV-Vis	ultraviolet-visible
ROS	reactive oxygen species
